# Infliximab, an anti-TNF-alpha agent, improves left atrial abnormalities in patients with rheumatoid arthritis: preliminary results

**DOI:** 10.5830/CVJA-2014-036

**Published:** 2014

**Authors:** Çetin Süha, Vural Mustafa Gökhan, Yeter Ekrem, Doğan Mehmet, Keskin Göksal, Öztürk Mehmet Akif

**Affiliations:** Department of Cardiology, 29 Mayis Hospital, Ankara, Turkey; Department of Cardiology, Dişkapi Research and Education Hospital, Ankara, Turkey; Department of Cardiology, Dişkapi Research and Education Hospital, Ankara, Turkey; Department of Cardiology, Dişkapi Research and Education Hospital, Ankara, Turkey; Department of Internal Medicine, Dişkapi Research and Education Hospital, Ankara, Turkey; Department of Rheumatology, School of Medicine, Gazi University, Ankara, Turkey

**Keywords:** electrocardiography, infliximab, left atrium, rheumatoid arthritis, speckle tracking echocardiography

## Abstract

**Background:**

Rheumatoid arthritis (RA) is associated with increased cardiovascular morbidity and mortality. In the current prospective study, we addressed the impact of RA on left atrial (LA) function and electrical remodelling. Further, we tried to demonstrate the effects of infliximab, an anti-TNFalpha agent, on echocardiographical LA abnormality in RA patients with preserved left ventricular (LV) ejection fraction.

**Methods:**

We compared 38 female RA patients without clinical evidence of heart disease and 30 female controls without RA and clinical evidence of heart disease. Further, we compared RA patients receiving infliximab and increasing doses of prednisolone over a three-month period. At baseline and post treatment, this study assessed (1) LA and LV parameters using conventional and speckle tracking echocardiography (STE), and (2) electrocardiographic P-wave changes.

**Results:**

The values of C-reactive protein (CRP), isovolumic relaxation time (IVRT), A wave, and deceleration time (DT) were significantly higher in RA patients compared to the control group (*p* < 0.05), whereas E/E′ and E/A values were found to be lower (*p* < 0.05) in RA patients. E/E′ values were lower in prednisolone- compared to infliximab-treated patients (*p* < 0.05). After three months of infliximab and prednisolone treatment, CRP and disease activity score (DAS 28) values decreased in both groups (*p* < 0.05), and Duke activity status index (DASI) increased (*p* < 0.05). Maximal left atrial volume index (LAVI_max_), pre-contraction left atrial volume index (LAVI_preA_) and maximum P wave (P_max_) of the RA patients were higher compared to the control group (*p* < 0.05), whereas LA global strain was found to be lower (*p* < 0.05). There was no difference in P_max_ values between groups before and after the treatment period. E/E′, LAVI_max_ and LAVI_preA_ values of infliximab-treated patients decreased and LA global strain increased after three months of therapy compared to baseline (*p* < 0.05). At baseline in both treatment groups, E/E′ and LA global late diastolic strain rate were lower in prednisolone-compared to infliximab-treated patients (*p* < 0.05).

**Conclusion:**

There was echocardiographic LA abnormality in these RA patients. In this patient group there was also a meaningful increase in maximum P wave assessed by electrocardiography. Infliximab therapy for a period of three months improved LA abnormality.

## Abstract

Rheumatoid arthritis (RA) is a systemic autoimmune disease affecting about 1% of the population.^1^ It is also characterised by an excess risk of cardiovascular disease and mortality, probably via chronic systemic inflammation.^2^ TNF-alpha represents the major inflammatory cytokine in RA patients.^3^ Apart from being the major promoter and regulator of the inflammatory cascade resulting in joint damage, it may mediate cardiac injury through a variety of biological mechanisms.^4^ Treatment of RA with anti-TNF-alpha agents such as infliximab has been shown to be effective in reducing signs and symptoms of the disease and in preventing joint damage,^5^-^7^ but their impact on cardiovascular disease, especially in RA patients with preserved LV function (EF ≥ 50) remains controversial.

In this prospective study we tried to elucidate (1) whether myocardial abnormality as assessed by echocardiography is present in RA patients compared to normal controls, (2) the electrocardiographic P-wave changes in RA patients, and (3) the impact of infliximab treatment on left ventricular (LV) and left atrial (LA) echocardiographic parameters in comparison to treatment with corticosteroids. We used conventional and speckle tracking echocardiography (STE), a novel method for the evaluation of myocardial abnormality.

## Methods

Thirty-eight female patients (age 52.1 ± 11.1 years) with RA diagnosed by revised American Rheumatism Association criteria, who had an inadequate response to disease-modifying antirheumatic drugs (DMARDs) and corticosteroids were recruited from the RA out-patient section of the Rheumatology Unit, Ministry of Health, Dişkapi Yildirim Beyazit Research and Education Hospital, Ankara, Turkey, between January 2011 and January 2012.^8^ The control group consisted of 30 female patients without RA (age 50.7 ± 3.4 years).

Patients of the RA group were on methotrexate 15 mg once per week and prednisolone 5–7.5 mg once daily. The patients had occasionally been treated with non-steroidal anti-inflammatory drugs within the previous six months. The disease activity score (DAS 28), which utilises C-reactive protein (CRP) level, and the visual analogue score of wellbeing plus the number of tender and swollen joints, was used in order to evaluate the activity of RA.^9^ We used the Duke activity status index (DASI), a brief self-administered questionnaire designed to estimate the patient’s exercise capacity in metabolic equivalents (METs).^10^

Out of 38 RA patients, 20 subjects (age 53.4 ± 13.5 years) additionally received infliximab treatment (initially 3 mg/kg; the same dose two and six weeks after the first infusion, and thereafter the same dose every eight weeks) for three months. The remaining 18 patients in the RA group were treated with prednisolone in increasing doses in accordance with standard clinical practice.

Patients were examined in the out-patient clinic monthly to assess clinical status and compliance with therapy. None of the patients had had ischaemic or arrhythmic events during the previous year. The RA patients’ biochemical parameters, and echocardiographic LA and LV function were measured at baseline and after three months of infliximab and prednisolone therapy. Electrocardiographic P waves were evaluated. The control subjects had a single baseline measurement of the examined parameters. The study protocol was approved by the local ethics committee.

## Conventional echocardiography

All echocardiographic examinations were performed by a single experienced observer blinded to clinical and laboratory data. Transthoracic echocardiography studies were performed using a commercially available ultrasound system with a 2.5–3.5 MHz transducer (ie33, Phillips Medical System, Bothell, Washington, USA). Patients lay at rest in the left lateral decubitus position, and apical four-chamber and parasternal views of the LA and LV were obtained at end-expiratory apnoea. Three cardiac cycles were stored from each view in ciné-loop format for subsequent off-line analysis by an investigator blinded to the patients’ data. Speckle tracking analysis was performed off-line by commercially available software QLAB 6.0 (Phillips Medical System, Bothell, Washington, USA).

We measured the following parameters from crosssectional echocardiographic images of the cardiac chambers. (1) End-diastolic interventricular septum thickness (IVS), end-diastolic posterior wall thickness (PW) of the LV, (2) end-diastolic volume (EDV), end-systolic volume (ESV) and ejection fraction of the LV using the modified Simpson’s method,^11^ and (3) maximal (LAVI_max_), pre-contraction (LAVI_preA_) and minimal (LAVI_min_) LA volumes were measured just before mitral valve opening, at the beginning of the P wave, and at mitral valve closure.

All LA volumes were determined using the modified Simpson’s method. LA volume indices were calculated by dividing the LA volumes by the body surface area.^12^

Pulsed-wave Doppler of transmitral LV inflow was performed in the apical four-chamber view, with the sample volume placed at the level of the mitral valve tips, and Doppler variables were analysed during three consecutive beats. The following measurements of LV diastolic function were determined: peak early (E) and late (A) diastolic mitral flow velocity and their ratio E/A, early diastolic mitral annular velocity (E’), late diastolic mitral annular velocity (A′), deceleration time of the E wave, and LV isovolumic relaxation time (IVRT).^13^ The E/E′ ratio was used as an index of LV filling pressures.

## Speckle tracking echocardiography

The methods of image acquisition and post-processing of strain and strain rate measurements with speckle tracking have been described previously.^14^ All images were obtained at a frame rate of 50 to 80 frames per second.

Briefly, the observer traced the endocardial and epicardial borders of the LA on an end-systolic frame and the software automatically tracked the border on the subsequent frames. Adequate tracking can then be verified in real-time and corrected by adjusting the region of interest or manually correcting the border to ensure optimal tracking. The aortic valve closure measured by Doppler has been identified as end-systole. The software is able to represent abnormality in time–strain graphs where it is possible to identify the different phases of the cardiac cycle.

## Electrocardiography

After a 20-minute resting period in the supine position, all subjects underwent a 12-lead ECG recording at a paper speed of 50 mm/s and 2 mV/cm. The P-wave duration was measured manually in all simultaneously recorded 12 leads of the surface ECG by one of the investigators blinded to the study hypothesis. In each lead the mean values for the three complexes were calculated. For greater accuracy, the measurements were performed with callipers and a magnifying lens, as described by previous investigators.^15^,^16^

The onset of the P wave was defined as the point of first visible upward departure from baseline for positive waveforms, and as the point of first downward departure from baseline for negative waveforms. The return to baseline was considered to be the end of the P wave. P_max_ measured in any of the 12 leads of the surface ECG was used as the longest atrial conduction time. The difference between P_max_ and minimum P wave (P_min_) was calculated and defined as P-wave dispersion (Pd = P_max_ – P_min_).^17^

## Laboratory assays

CRP was measured using routine methods. IgM rheumatoid factor (RF) was measured by means of immunonephelometry using the quantitave N Latex RF system (Dade Behring, Marburg, Germany) with RF titers of > 15 IU/ml being considered positive. Serum levels of total cholesterol, low-density lipoprotein (LDL) cholesterol, high-density lipoprotein (HDL) cholesterol and creatinine were determined using an auto-analyser under fasting conditions on the same day as the other evaluations.

## Statistical analysis

Propensity scores, a methodology that can be used to compare the effectiveness of different treatments and to examine whether patients included in the two treatment groups were adequately balanced for atherosclerosis, between infliximab- and prednisolone-treated patients, were compared using a two-tailed *t*-test. Categorical data were compared by contingency tables, and between each treatment group and normal controls by the χ^2^ test or Fisher exact test when five patients or fewer were included in each cell.

Continuous variables were tested for normality using the Kolmogorov–Smirnov test. Normally distributed variables are given as mean and standard deviation (SD). Spearman correlation analysis was used to determine bivariate correlations. Because biomarkers had a non-normal distribution, data are expressed as median (interquartile range) and were analysed after transformation into ranks.

Analysis of variance (ANOVA) for repeated measurements was applied to compare the effects of infliximab versus prednisolone, with measurements at baseline and three months post treatment used as a within-subject factor, and type of treatment as between-subject factor. The *F*- and *p*-values of interaction between time measurement of the examined markers and type of treatment were calculated.

Post hoc comparisons were performed within Bonferroni’s correction. Comparisons between controls and each treatment group at baseline or three months were performed using the unpaired *t*-test (normally distributed variables) and Mann–Whitney test (non-normally distributed variables). Statistical significance was considered as *p* < 0.05. All statistical analysis was performed using SPSS for Windows (release 15.0, SPSS Inc, Chicago, Illinois).

## Results

The demographic, clinical, biochemical and conventional echocardiographic parameters are given in Tables 1 and 2. All RA patients were seropositive. The mean DAS was 6.4 ± 0.7 and the disease duration was 85.7 ± 66.8 months.

**Table 1:** Clinical and biochemical characteristics of the study population

	*Controls (n = 30)*	*RA patients (n = 38)*	*Infliximab-treated patients (n = 20)*	*Prednisolone-treated patients (n = 18)*	p-value*	p*-value^#^*
DAS-28	–	6.4 ± 0.7	6.4 ± 0.5	6.1 ± 0.8	–	0.06
Disease duration (months)	–	85.7 ± 66.8	98.4 ± 77.4	71.6 ± 51.1	–	0.21
RF (mg/dl)	–	226.8 (25.2–66.1)	194.4 (25.2–321.8)	165.8 (30.5–366.1)	–	0.57
Age (years)	50.7 ± 3.4	52.1 ± 11.1	53.4 ± 13.5	50.7 ± 7.6	0.52	0.44
Body mass index (kg/m^2^)	30.5 ± 3.7	30.5 ± 5.5	31.0 ± 5.9	29.9 ± 5.2	0.91	0.70
Obesity (%)	7 (23)	5 (13)	2 (10)	3 (16)	0.21	0.47
Hypertension (%)	10 (33)	17 (44)	9 (45)	8 (44)	0.24	0.63
Current smoking (%)	8 (26)	5 (13)	2 (10)	3 (16)	0.13	0.32
Dyslipidaemia (%)	8 (26)	8 (21)	3 (15)	5 (27)	0.39	0.56
Diabetes mellitus (%)	2 (6)	8 (21)	4 (20)	4 (22)	0.09	0.24
Medication						
RAAS blocker (%)	5 (16)	11 (28)	6 (30)	5 (27)	0.18	0.48
β-blocker (%)	5 (16)	4 (10)	1 (5)	3 (16)	0.34	0.43
CaCh blocker (%)	4 (13)	4 (10)	3 (15)	1 (5)	0.5	0.62
Statin (%)	7 (23)	4 (10)	2 (10)	2 (11)	0.13	0.36
SBP (mmHg)	121.6 ± 9.8	124.7 ± 13.9	122.1 ± 14.4	127.4 ± 13.2	0.33	0.24
DBP (mmHg)	79.0 ± 6.6	78.7 ± 8.9	75.5 ± 9.1	82.2 ± 7.5	0.92	0.12
HR (beats/min)	70.6 ± 6.3	74.4 ± 10.5	74.9 ± 12.2	71.1±7.7	0.45	0.5
Total cholesterol (mg/dl)	171.1 ± 29.7	163.0 ± 24.6	170.3 ± 20.2	166.5 ± 10.8	0.4	0.74
HDL cholesterol (mg/dl)	39.1 ± 8.1	41.3 ± 11.3	40.4 ± 12.9	43.7 ± 9.5	0.36	0.53
LDL cholesterol (mg/dl)	112.4 ± 16.4	117.2 ± 26.6	115.6 ± 25.4	119.0 ± 22.9	0.44	0.76
Glucose (mg/dl)	96.7 ± 10.7	98.4 ± 15.9	102.3 ± 13.1	98.6 ± 10.0	0.77	0.47
Creatinine (mg/dl)	0.8 ± 0.1	0.8 ± 0.2	0.8 ± 0.1	0.8 ± 0.1	0.62	0.82
CRP (mg/dl)	1.2 (0.6–4.3)	20.4 (8.0–34.9)	20.4 (10.5–34.9)	17.6 (8.0–33.5)	< 0.01	0.16

Values are expressed as mean ± SD. Values for CRP and RF are median and interquartile range. *For comparisons between RA patients and control group. ^#^For comparisons between infliximab- and prednisolone-treated patients. DAS-28 = disease activity score, RF = rheumatoid factor, RAAS = renin–angiotensin–aldosteron system, CaCh = calcium channel, SBP = systolic blood pressure, DBP = diastolic blood pressure, HR = heart rate, CRP = C-reactive protein.

**Table 2:** Conventional echocardiographic characteristics of the study population

	*Controls (n = 30)*	*RA patients (n = 38)*	*Infliximab-treated patients (n = 20)*	*Prednisolone-treated patients (n = 18)*	p-value*	p*-value^#^*
LV EDV (ml)	80.1 ± 10.1	83.2 ± 6.1	85.7 ± 6.6	83.9 ± 4.7	0.74	0.2
LV ESV (ml)	29.3 ± 5.6	28.8 ± 6.2	28.3 ± 4.2	29.2 ± 5.3	0.49	0.51
LV EF (%)	64.6 ± 4.1	64.2 ± 3.0	63.8 ± 4.0	64.1 ± 3.3	0.35	0.7
IVS (mm)	9.5 ± 0.6	9.7 ± 0.5	9.7 ± 1.7	9.5 ± 2.5	0.39	0.06
PW (mm)	8.5 ± 0.6	8.5 ± 0.4	8.6 ± 0.6	8.5 ± 0.8	0.91	0.82
LAV (ml)	38.7 ± 4.8	39.8 ± 4.0	39.4 ± 3.3	40.4 ± 4.6	0.29	0.43
S′ (cm/s)	7.6 ± 2.9	7.3 ± 3.2	7.2 ± 1.8	7.4 ± 2.5	0.16	0.1
E′ (cm/s)	9.0 ± 3.1	8.6 ± 2.1	7.9 ± 2.5	8.3 ± 1.9	0.18	0.09
E/ E′	7.8 ± 2.0	9.0 ± 2.6	9.4 ± 3.9	8.5 ± 2.5	< 0.05	< 0.05
IVRT (ms)	86.8 ± 5.5	93.7 ± 10.4	96.7 ± 10.8	90.3 ± 10.1	< 0.01	0.56
E (cm/s)	78.1 ± 12.5	76.9 ± 7.7	76.1 ± 7.4	77.9 ± 8.2	0.13	0.48
A (cm/s)	49.5 ± 9.3	69.3 ± 10.6	67.5 ± 9.9	71.3 ± 11.2	< 0.001	0.28
E/A	1.4 ± 0.2	1.14 ± 0.25	1.1 ± 0.2	1.1 ± 0.2	< 0.001	0.66
DT (ms)	179.8 ± 25.5	199.3 ± 32.6	199.1 ± 35.3	198.6 ± 30.3	< 0.001	0.86

Values are expressed as mean ± SD. *For comparisons between RA patients and control group. ^#^For comparisons between infliximab- and prednisolone-treated patients. LV = left ventricle, EDV = end-diastolic volume, ESV = end-systolic volume, EF = ejection fraction, IVS = interventricular septum, PW = posterior wall, LAV = left atrial volume, IVRT = isovolumic relaxation time, DT = deceleration time.

Age, cardiac medication, cardiovascular risk factors, systolic and diastolic blood pressure, heart rate, and cholesterol, fasting glucose and creatinine levels were similar between the control group and the RA patients and also in the infliximab- and prednisolone-treated patients. Therefore our patients had similar characteristics regarding risk factors for atherosclerosis.

Control group patients had normal electrocardiograms, transthoracic echocardiography and treadmill tests. Baseline CRP, DAS 28 and RF values showed significant differences. None of the subjects was excluded from the study because of adverse effects or discontinuation of therapy.

On transthoracic echocardiography there was no pericardial effusion or significant valvular heart disease. Parameters showing diastolic function such as LV diastolic filling pressure (E/E′), A-wave values, deceleration time (DT) and isovolumic relaxation time (IVRT) were significantly higher in the RA patients. E/A ratio showed a significant reduction (*p* < 0.05). E/E′ was found to be decreased in prednisolone- compared to infliximab-treated patients (*p* < 0.05).

Significant improvement in RA parameters, as assessed by CRP, DAS 28 and DASI were achieved in both treatment groups (*p* < 0.05) (Table 3).

**Table 3:** Effects of infliximab treatment on RA parameters versus prednisolone-treated patients

	*Infliximab-treated RA patients (n = 20)*			*Prednisolone-treated RA patients (n = 18)*		
	*Baseline*	*3-month*	p*-value*	*Baseline*	*3-month*	p*-value*
CRP (mg/dl)	19.9 ± 6.4	4.7 ± 1.2	< 0.05	17.5 ± 7.5	5.6 ± 2.2	< 0.05
DAS-28	6.4 ± 1.0	5.4 ± 1.1	< 0.05	7.1 ± 0.7	6.2 ± 1.0	< 0.05
DASI	3.9 ± 0.8	6.8 ± 0.5	< 0.05	3.6 ± 1.0	6.1 ± 0.9	< 0.05

Values are expressed as mean ± SD. CRP = C-reactive protein, DAS-28 = disease activity score 28, DASI = Duke activity status index.

Table 4 shows LA echocardiographic and ECG parameters between RA patients and controls: LAVI_max_ and LAVI_preA_ revealed a significant increase in comparison with the control group (*p* < 0.05). As assessed by two-dimensional (2D) STE, the global left atrial strain showed a significant impairment in the RA patients (*p* < 0.05). Electrocardiographically we evaluated the P-wave durations, P_max_, P_min_, and Pd. In the RA patients, P_max_ was significantly higher compared to the control group (*p* < 0.05). There were no differences in P_max_ values between the groups before and after the treatment period.

**Table 4:** Comparisons of left atrial echocardiographic and electrocardiographic parameters between rheumatoid arthritis patients and the control group

	*Controls (n = 30)*	*RA patients (n = 38)*	p*-value*
LAVI_max_ (ml/m^2^)	19.7 ± 3.9	23.4 ± 3.3	< 0.001
LAVI_preA_ (ml/m^2^)	15.1 ± 3.3	17.1 ± 3.4	< 0.01
LAVI_min_ (ml/m^2^)	10.9 ± 3.2	10.9 ± 2.6	0.915
P_max_ (ms)	83.3 ± 9.5	88.6 ± 9.0	< 0.05
P_min_ (msec)	41.3 ± 5.7	43.1 ± 7.3	0.255
P dispersion (msec)	42.0 ± 8.0	45.5 ± 6.4	0.056
LA global strain (%)	34.1 ± 10.1	28.3 ± 13.4	< 0.05
LA global systolic strain rate (/s)	1.8 ± 0.3	1.7 ± 0.3	0.053
LA global early diastolic strain rate (/s)	–1.2 ± 0.2	–1.2 ± 0.3	0.434
LA global late diastolic strain rate (/	–1.0 ± 0.3	–1.1 ± 0.2	0.492

Values are expressed as mean ± SD. LA = left atrium, LAVI = left atrial volume index, P = electrocardiographic P wave.

Tables 5 and 6 illustrate the effects of infliximab therapy on LA and LV echocardiographic and electrocardiographic parameters versus the prednisolone-treated patients. Baseline conventional and 2D STE parameters were similar between the two treatments groups. Only E/E′ ratio and LA global late diastolic strain rate showed some significant differences (*p* < 0.05). There was a significant improvement in E/E′, LAVI_max_, LAVI_preA_ and LA global strain values in the infliximab-treated patients (*p* < 0.05).

**Table 5:** Effects of infliximab therapy on conventional echocardiographic parameters versus prednisolone-treated patients

	*Infliximab-treated RA patients (n = 20)*			*Prednisolone-treated RA patients (n = 18)*			
	*Baseline*	*3-month*	p*-value*	*Baseline*	*3-month*	p*-value*	p*-value^#^*
LV EDV (ml)	81.7 ± 6.6	79.7 ± 6.9	0.411	83.9 ± 4.7	85.4 ± 9.8	0.868	0.2
LV ESV (ml)	28.3 ± 4.2	27.2 ± 3.6	0.14	29.2 ± 5.3	27.9 ± 2.6	0.557	0.5
LV EF (ml)	63.8 ± 4.0	64.6 ± 2.8	0.096	64.1 ± 3.3	64.9 ± 2.8	0.35	0.71
IVS (mm)	9.7 ± 1.7	9.5 ± 3.9	0.267	9.5 ± 2.5	9.4 ± 4.6	0.316	0.06
PW (mm)	8.6 ± 0.6	8.3 ± 0.4	0.056	8.5 ± 0.8	8.7 ± 0.4	0.381	0.82
S′ (cm/sec)	7.2 ± 1.8	6.8 ± 0.8	0.258	7.4 ± 2.5	7.6 ± 0.6	0.369	0.1
E′ (cm/sec)	7.9 ± 2.5	9.0 ± 1.3	0.181	8.3 ± 1.9	7.8 ± 2.4	0.6	0.09
E/E′	9.4 ± 3.9	8.0 ± 1.4	< 0.01	8.5 ± 2.5	8.8 ± 2.4	0.379	< 0.05
E (cm/s)	76.1 ± 7.4	76.0 ± 11.8	0.979	77.9 ± 8.2	79.5 ± 11.7	0.493	0.476
A (cm/s)	67.5 ± 9.9	66.0 ± 7.7	0.221	71.3 ± 11.2	70.2 ± 9.8	0.407	0.284
E/A	1.1 ± 0.2	1.1 ± 0.2	0.758	1.1 ± 0.2	1.1 ± 0.3	0.184	0.656
DT (ms)	199.1 ± 35.3	203.0 ± 34.1	0.609	198.6 ± 30.3	211.5 ± 22.4	0.055	0.858
IVRT (ms)	96.7 ± 10.8	95.3 ± 16.9	0.626	90.3 ± 10.1	90.3 ± 16.8	0.99	0.056

Values are expressed as mean ± SD. ^#^For comparisons between infliximab- and prednisolone-treated patients at baseline. LV = left ventricle, EDV = end-diastolic volume, ESV = end-systolic volume, EF = ejection fraction, IVS = interventricular septum, PW = posterior wall, DT = deceleration time, IVRT = isovolumic relaxation time.

**Table 6:** Effects of infliximab therapy on left atrial echocardiographic and electrocardiographic parameters versus prednisolone-treated patients

	*Infliximab-treated RA patients (n = 20)*			*Prednisolone-treated RA patients (n = 18)*			*p-value^#^*
	*Baseline*	*3-month*	p*-value*	*Baseline*	*3-month*	p*-value*	
LAVI_max_ (ml/m^2^)	23.1 ± 2.9	21.9 ± 2.3	< 0.05	23.7 ± 3.7	23.3 ± 2.1	0.453	0.435
LAVI_preA_ (ml/m^2^)	16.8 ± 2.7	15.8 ± 2.1	< 0.01	17.5 ± 3.0	17.6 ± 3.1	0.892	0.369
LAVI_min_ (ml/m^2^)	11.4 ± 2.2	11.0 ± 2.1	0.082	10.4 ± 2.9	10.1 ± 1.5	0.368	0.066
P_max_ (ms)	86.0 ± 8.8	82.0 ± 10.5	0.072	91.6 ± 8.5	87.7 ± 10.6	0.09	0.052
P_min_ (ms)	42.5 ± 7.8	41.0 ± 12.5	0.505	43.8 ± 6.9	42.7 ± 14.4	0.749	0.568
P dispersion (ms)	43.5 ± 7.4	41.0 ± 7.1	0.056	47.7± 4.2	45.0 ± 7.8	0.172	< 0.05
LA global strain (%)	25.4 ± 10.6	30.4 ± 2.6	< 0.05	31.5 ± 14.7	36.3 ± 6.9	0.21	0.181
LA global systolic strain rate (ms)	1.6 ± 0.3	1.7 ± 0.2	0.073	1.7 ± 0.3	1.7 ± 0.4	0.536	0.59
LA global early diastolic strain rate (/s)	–1.1 ± 0.3	–1.2 ± 0.3	0.098	–1.2 ± 0.3	–1.2 ± 0.2	0.751	0.596
LA global late diastolic strain rate (/s)	–1.2 ± 0.2	–1.2 ± 0.2	0.567	–1.0 ± 0.2	–1.1 ± 0.2	0.367	< 0.01

Values are expressed as mean ± SD. ^#^For comparisons between infliximab- and prednisolone-treated patients at baseline. LA = left atrium, LAVI = left atrial volume index.

## Discussion

In this prospective, preliminary study, we showed echocardiographic LA abnormalities in RA patients in comparison to the control group. Furthermore, there was an improvement in LA abnormalities in the RA patients who were treated with infliximab in comparison to the prednisolone-treated group.

There is substantial evidence that RA is associated with increased cardiovascular morbidity and mortality.^18^ Cardiovascular manifestations of RA include atherosclerosis, myocardial infarction, heart failure and cerebrovascular disease.^19^ It is becoming increasingly apparent that inflammation mediators are strongly linked to this excess risk of cardiovascular disease and mortality.^20^

The impairment of coronary microcirculation may compromise myocardial perfusion and cause LV systolic and diastolic dysfunction.^21^,^22^ Additionally, interstitial fibrosis caused by cytokine-induced fibroblast activity, and collagen deposition in the heart muscle are present in RA.^23^ Previous studies^24^,^25^ also described reduced myocardial deformation markers assessed by STE in addition to abnormal tissue Doppler imaging (TDI) parameters in RA patients compared to controls.

As a further finding, there was a significant increase in P_max_ in the ECG of RA patients compared to the control group. The increase in atrial strain, as well as dilatation and fibrosis bring about a heterogenous and different conduction in the atrial myocardium.^26^-^28^ Such pathophysiological changes can trigger atrial re-entry, thus playing an important role in the development of atrial fibrillation.^26^

One of the most important cytokines implicated in the progression of chronic heart failure is TNF-alpha.^20^ Although treatment with anti-TNF-alpha agents represents a major advance in the treatment of rheumatic disease, its impact on cardiovascular risk, especially in RA patients with preserved LV function (EF ≥ 50), remains controversial.

In a study by Santos *et al.* there was a decrease in cardiac output and stroke volume in RA patients without clinical and echocardiographical evidence of previous cardiac dysfunction.^29^ On the other hand, Listing et al. showed that therapy with anti-TNF-alpha agents is more likely to be beneficial than harmful with regard to the risk of heart failure.^30^

**Fig. 1.**
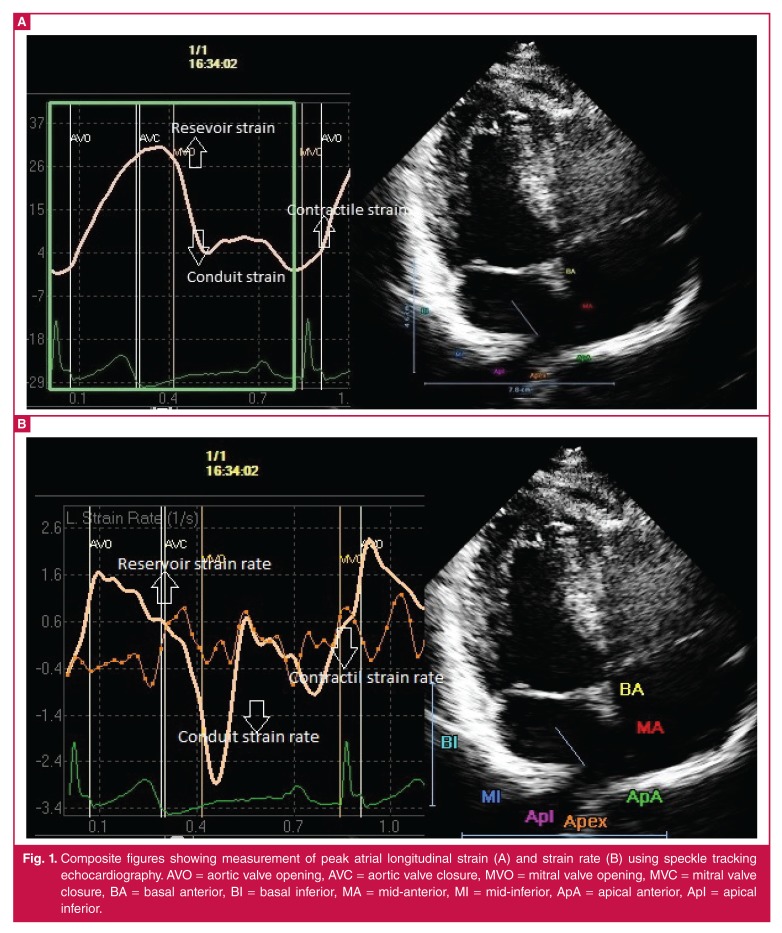
Composite figures showing measurement of peak atrial longitudinal strain (A) and strain rate (B) using speckle tracking echocardiography. AVO = aortic valve opening, AVC = aortic valve closure, MVO = mitral valve opening, MVC = mitral valve closure, BA = basal anterior, BI = basal inferior, MA = mid-anterior, MI = mid-inferior, ApA = apical anterior, ApI = apical inferior.

In the present study, we showed an improvement in LA global strain and volume index parameters in patients who were treated with infliximab, a monoclonal antibody against TNF-alpha. We used conventional echocardiography and STE. STE is an imaging technique, in which ultrasound speckles within the image are tracked, and strain is derived from the displacement of speckles relative to each other.^31^ This new modality enables accurate and reliable measurements of both global and regional myocardial strain and strain rates without the confounding effects of angle dependency (Fig. 1).^32^

To the best of our knowledge, this is the first study evaluating the effects of infliximab on LA function in RA patients. There are only limited data on the pathophysiological mechanisms in this regard.

In our study, significant improvement in LA global strain and volume indices after infliximab treatment was clearly demonstrated. In addition, there was also a remarkable improvement in parameters of LV diastolic dysfunction as shown by ‘better’ E/E′ values following infliximab treatment. E/E′ value, which is used as an index of LV filling pressures, correlates strongly with LV diastolic dysfunction.^33^-^35^

There are two possible explanations for these findings. First, we may speculate that LV diastolic dysfunction in patients with RA may be a result of inflammation-induced endothelial dysfunction, a common finding in this patient group.^21^,^22^ Anti-TNF-alpha agents may restore endothelial function in these patients, which in turn reduces LV diastolic dysfunction and leads to ‘relief’ of LA.^36^,^37^ Second, as assessed with magnetic resonance imaging, LA strain has been related to LA structural remodelling and fibrosis of the atrial wall.^38^ By reducing inflammation mediators with anti-TNF-alpha agents, the fibroblast activity and collagen deposition in the LA myocardium may be diminished, which consequently could lead to improvement in LA function.

There are some limitations to this study. The first was the rather small number of RA patients in each subgroup, which limited the power of comparison. Further studies with larger numbers are needed to assess the effects of infliximab on stain and strain rate parameters of LA. The second limitation was the duration of treatment, which was only three months. The impact of the medication on cardiac function could have been much more impressive with a longer period of treatment.

## Conclusion

In this study, we demonstrated significant echocardiographical LA abnormalities in RA patients compared with the control group, in the presence of preserved LV systolic function. The early detection of myocardial abnormalities by conventional echocardiography and STE may be useful for better clinical assessment and treatment of cardiovascular disease. Furthermore, we showed a meaningful increase in P_max_ in the ECG of RA patients, which may contribute to the development of supraventricular arrhythmias. It was also found that patients with RA and preserved ejection fraction had significant improvement in LA function with anti-TNF-alpha treatment compared to patients treated with prednisolone.
